# What has been the progress in addressing financial risk in Uganda? Analysis of catastrophe and impoverishment due to health payments

**DOI:** 10.1186/s12913-020-05500-2

**Published:** 2020-08-12

**Authors:** Brendan Kwesiga, Tom Aliti, Pamela Nabukhonzo, Susan Najuko, Peter Byawaka, Justine Hsu, John E. Ataguba, Grace Kabaniha

**Affiliations:** 1 World Health Organization, Health Systems Cluster, Nairobi, Kenya; 2 Ministry of Health, Planning Department, Kampala, Uganda; 3 Uganda Bureau of Statistics, Kampala, Uganda; 4 World Health Organization, Economic Analysis Cluster, Geneva, Switzerland; 5 Health Economics Unit, School of Public Health and Family Medicine, University of Cape Town, Cape Town, South Africa; 6 World Health Organization, Health Systems Cluster India Country Office, New Delhi, India

**Keywords:** Financial protection, Uganda, Universal health coverage, Health financing, Health, Trends, Impoverishment, Catastrophic health payments

## Abstract

**Background:**

Monitoring progress towards Universal Health Coverage (UHC) requires an assessment of progress in coverage of health services and protection of households from the impact of direct out-of-pocket payments (i.e. financial risk protection). Although Uganda has expressed aspirations for attaining UHC, out-of-pocket payments remain a major contributor to total health expenditure. The aim of this study is to monitor progress in financial risk protection in Uganda.

**Methods:**

This study uses data from the Uganda National Household Surveys for 2005/06, 2009/10, 2012/13 and 2016/17. We measure financial risk protection using catastrophic health care payments and impoverishment indicators. Health care payments are catastrophic if they exceed a set threshold (i.e. 10 and 25%) of the total household consumption expenditure. Health payments are impoverishing if they push the household below the poverty line (the US$1.90/day and Uganda’s national poverty lines). A logistic regression model is used to assess the factors associated with household financial risk.

**Results:**

The results show that while progress has been made in reducing financial risk, this progress remains minimal, and there is still a risk of a reversal of this trend. We find that although catastrophic health payments at the 10% threshold decreased from 22.4% in 2005/06 to 13.8% in 2012/13, it increased to 14.2% in 2016/17. The percentage of Ugandans pushed below the national poverty line (US$1.90/day) has decreased from 5.2% in 2005/06 to 2.7% in 2016/17. The distribution of both catastrophic health payments and impoverishment varies across socio-economic status, location and residence. In addition, certain household characteristics (poverty, having a child below 5 years and an adult above 60 years) are more associated with the lack of financial risk protection.

**Conclusion:**

There is need for targeted interventions to reduce OOP, especially among those affected so as to increase financial risk protection. In the short-term, it is important to ensure that public health services are funded adequately to enable effective coverage with quality health care. In the medium-term, increased reliance on mandatory prepayment will reduce the burden of OOP health spending further.

## Background

Uganda has expressed aspirations to attain Universal Health Coverage (UHC) [1]—a key component of the Sustainable Development Goals (SDGs) agenda. UHC is about ensuring that the population has access to needed health services that are of adequate quality to be effective, without facing any financial risk that results from paying out-of-pocket (OOP) for health services [2–4]. Many countries, including Uganda, still face challenges to achieving UHC [5]. For example, in Uganda, OOP payments for health services are still dominant, contributing up to 40% of Uganda’s total health expenditure [6], even though user fees/cost-sharing in government facilities have been abolished since 2001. This phenomenon presents a paradox [7].

Safeguarding households from incurring financial risks and minimising the risk of falling into poverty, through ensuring that households’ consumption of other basic needs such as food and shelter are not compromised due to direct OOP payments is critical [3]. This is even more important for a country like Uganda, where more than a quarter of the population (about 10 million Ugandans) in absolute poverty [8] and more than 40% of the population remains vulnerable to economic shocks [9]. This situation raises an urgent need to implement health financing strategies that address the burden of OOP payments.

Financial resources to Uganda’s health sector remain very inadequate. Government per capita health expenditure averaged US$9 in the past ten years [10]. This is grossly below the US$84 recommended to provide a minimum health care package [11] or the US$271 per capita estimated for achieving UHC by 2030 [12]. Furthermore, the proportion of the health budget allocated to the health sector (an indication of the level of prioritisation of the sector) declined to an average of about 7% in the period between 2015 and 2019 from about 9% in 2010–2015 [10]. Low levels of public health financing led to the health sector, increasingly relying on OOP health spending and external resources [6].

It is not surprising that Uganda’s Health Financing Strategy 2015–2025 identifies the need to address the current burden of OOP payments [13]. In designing such strategies to address financial risk in the context of moving towards UHC, there is a need for up-to-date country-specific evidence on the extent and distribution of the burden of OOP health spending across the population.

Adequately monitoring of UHC at both the global and country levels is required to harness the benefits of efforts towards UHC. To achieve this goal, the World Bank Group together with the World Health Organization (WB/WHO) have developed a framework for monitoring and evaluation of progress towards UHC [14]. This framework identifies two major indicators for monitoring financial risk protection—catastrophic health payments and impoverishing health expenditures. The catastrophic health payments indicator looks at the extent to which the share of OOP health payments in total household consumption expenditure does not compromise consumption of other household basic needs while the impoverishment indicator looks at the extent to which OOP payments increase both the incidence and intensity of poverty [14]. This framework also emphasizes the need to use several equity dimensions to monitor progress towards UHC.

Previous studies have analyzed financial risk protection in Uganda’s health system [15, 16] using dated datasets. However, they do not show the trend in financial risk protection in Uganda using recent datasets, which is critical for monitoring progress towards the UHC goals. The objective of this paper is to present an updated assessment of financial risk protection in health using indicators from the WB/WHO framework. The paper also presents the trend from 2005/6 to 2016/17 to track Uganda’s progress towards UHC. This study provides baseline information on the extent of financial risk protection in health as Uganda plans to roll out interventions including a national health insurance to decrease the reliance on OOP payments for health services and ensure financial risk protection for all.

## Methods

### Data

The Uganda integrated household survey, known as the Uganda National Household Survey (UNHS) is the main data for the analysis in this paper. The UNHS is undertaken every three or four years by the Uganda Bureau of Statistics (UBOS) and collects individual and household level data about socioeconomic characteristics, health status, health-seeking behaviour, and household expenditures including health expenditure. This paper uses the UNHS data for the years 2005/6, 2009/10, 2012/13 and 2016/17 containing data on 7400, 6887, 7500, and 17,320 households, respectively. Stata version 13.1 [17] is used for data analysis.

### Measurement of socio-economic status

Household consumption expenditure is used as the measure of socio-economic status as opposed to household income because the former is recommended as a more reliable measure of socioeconomic status in low-income countries like Uganda [18]. The construction of Uganda’s consumption expenditure aggregate is detailed elsewhere [19]. Adult equivalent household consumption expenditure is obtained by dividing total household consumption by an adjusted household size (equivalence scale). The equivalence scale for Uganda is based on estimated calorie requirements for different age groups [19]. The consumption for household members below 18 years weighs less than that for adults in adjusting household expenditures. This approach for estimating the adjusted household size has been used elsewhere to assess both the catastrophic and the impoverishing effects of OOP health spending [16]. It is important to point out that using the equivalence scales as opposed to per capita approach (dividing household consumption expenditure by household size) results into different estimates of catastrophe and impoverishing effects.

### Measurement of catastrophic health payments

Two thresholds are used to assess financial catastrophe from OOP health spending in this paper. A household’s OOP spending on health services is defined as catastrophic if it exceeds 10% or 25% of total household expenditure (or consumption) [20]. Indicators of the incidence (headcount) and intensity (the mean positive gap) are considered. Catastrophic health payments headcount represents the percentage of household whose OOP payments for health exceed 10% or 25% of total household expenditure. On the other hand, the mean positive gap indicates by how much the households exceed the chosen threshold for those that exceed.

### Household characteristics associated with catastrophic health payments

The factors that are associated with incurring catastrophic health expenditures are assessed using a logistic regression model.

$$ \mathrm{cata}=\alpha +\beta X+\varepsilon $$

where “cata” is the incidence of catastrophic health expenditures, cata = 1 for a household with catastrophic expenditures, and 0 otherwise. The vector of explanatory variables (*X*) includes equalised household size, the level of education, sex, employment status and marital status of the household head, location of household (rural or urban), presence of a child below 5 years and an adult above 60 years, and region of residence. The explanatory variables and expected signs are shown in Table 1.

**Table 1:** Explanatory variables for the logistic regression

Variables	Expected sign
Poverty (poor = 1, non-poor = 0)	+
Residence (urban = 1, rural = 0)	–
Region (reference = central)	+/−
Sex of household head (male = 1, female = 0)	+/−
Number of people in the household	+
Children below 5 years in the household (yes =1, no = 0)	+
Adults above 60 years in the household (yes = 1, no = 0)	+
Education of household head (reference: no formal education)	–
Employment (reference: formal employment)	–
Marital status (married = 1, not married = 0)	+/−

+: Positive, −: Negative, +/−: Indeterminate

### Measurement of impoverishment due to OOP health spending

Impoverishment from OOP spending on health services captures the extent by which OOP payment affects both the incidence and the depth/intensity of poverty across the population [20, 21]. Unlike the assessment of financial catastrophe, impoverishment headcount from paying OOP for health services estimates the difference between the percentage of the population below a defined poverty line before and after adjusting for the effect of OOP health payments [20]. The impoverishment gap from OOP spending is the difference in impoverishment gap (i.e. the extent to which an individual falls below the poverty line) before and after OOP health spending. The normalised impoverishment gap is computed by dividing the impoverishment gap by the poverty line—this is the impoverishment gap as a proportion of the poverty line.

Two poverty lines are used to assess impoverishment from OOP health spending. The first is the international poverty line (i.e. $1.90/per day based on 2011 Purchasing Power Parity (PPP).^1^ The second is Uganda’s national poverty line, which is region and location (urban/rural) specific. The average national poverty line is Shs. 29,505 but varies from Shs. 32,106 per month in the central region (urban) to Shs. 28,165 per month in the western region (rural). Uganda’s national poverty line is constructed based on the calorie requirement of household members and then adjusted for household non-food expenditures ([19]).

## Results

### Catastrophic health payments

Table 2 indicates the trend of the catastrophic headcount and the mean positive gap for two thresholds (10 and 25%). For both thresholds, there has been a decreasing pattern in terms of the catastrophic health payments headcount between 2005/06 and 2012/13. However, there was an increase between 2012/13 and 2016/17, irrespective of the thresholds. Concerning the mean positive gap, there has been a decreasing pattern for both thresholds, decreasing from 2005/06 to 2016/17.

**Table 2:** Household catastrophic health payments

Year	10%		25%	
	Headcount (%)	Mean positive gap (%)	Headcount (%)	Mean positive gap (%)
2005/06	22.4	11.5	5.9	13.1
2009/10	21.4	11.0	5.4	12.2
2012/13	13.8	8.9	2.6	10.9
2016/17	14.2	8.8	2.7	8.2

Source: Authors’ computation based on the UNHS 2005/06-2016/17

Table 3 shows catastrophic payments disaggregated by social-economic status, urban/rural location, and region of residence. The incidence of catastrophic health expenditure was higher among the richer quintiles when compared to the poorest quintile in the first three years. The reverse was true in 2016/17, where the poorer quintiles experienced a higher incidence of catastrophic payments. The incidence of catastrophic costs was much higher in the rural areas than in the urban areas in 2005/06 and 2009/10 with the pattern changing in 2012/13 and 2016/17. With regards to the regions, catastrophic health payments are highest in the Western and Central regions between 2005 and 2017.

**Table 3:** Disaggregation of catastrophic health expenditure (10% of total household expenditure)

Disaggregation variable	2005/06	2009/10	2012/13	2016/17
**Total**	22.4	21.4	13.8	14.2
**Socio-economic status quintiles**				
Poorest	18.9	17.2	9.6	7.7
Second poorest	20.4	18.9	10.0	13.7
Middle	24.2	21.5	12.6	14.1
Second richest	26.5	24.2	18.1	19.2
Richest	22.0	25.0	18.7	16.5
**Poverty Status**				
Non-poor	23.7	22.6	14.8	14.7
Poor	19.5	17.2	9.8	12.8
**Residence**				
Rural	23.5	21.7	13.5	15.3
Urban	16.2	19.5	14.9	13.0
**Region**				
Central	20.3	21.9	19.8	15.3
Eastern	21.1	21.6	9.1	13.0
Northern	20.2	18.3	13.1	13.9
Western	27.8	23.1	13.8	14.7

Source: Authors’ computation based on the UNHS 2005/06-2016/17 data

There are some household characteristics associated with an increased likelihood of catastrophic health payments. As shown in Table 4, the factors which are significantly associated (5% level of significance) with an increased likelihood of catastrophic health expenditures are having a child, and an elderly member in the household.

**Table 4:** Determinants of catastrophic health expenditure, 2016/17

Catastrophic health expenditure (10% of household expenditure)						
Independent Variables	Odds-ratio (OR)	SE.	z	P > z	[95% CI]	
**Poverty (poor = 1, non-poor = 0)**	0.4	0.0	−8.2	0.0	0.3	0.5
**Residence (urban = 1, rural = 0)**	0.8	0.1	−1.9	0.1	0.7	1.0
**Region (R = central)**						
Eastern	0.8	0.1	−1.5	0.1	0.7	1.1
Northern	0.9	0.1	−0.6	0.6	0.8	1.2
Western	0.8	0.1	−1.6	0.1	0.7	1.0
**Household size**	1.1	0.0	2.7	0.0	1.0	1.1
**Sex of household head** (male = 1, female = 0)	0.9	0.1	−1.0	0.3	0.7	1.2
**Employment** (R = formal)						
Casual/Subsistence	1.1	0.1	0.5	0.6	0.9	1.3
Unemployed	1.3	0.2	1.9	0.1	1.0	1.6
**Children below 5 (yes = 1, no = 0)**	1.3	0.1	3.1	0.0	1.1	1.5
**Adults above 60 (yes = 1, no = 0)**	1.4	0.2	3.3	0.0	1.2	1.7
**Education** (R = no formal education)						
Primary level	1.1	0.1	0.7	0.5	0.9	1.4
Secondary level	1.0	0.1	−0.2	0.9	0.7	1.3
Tertiary	0.7	0.2	−1.7	0.1	0.5	1.1
**Marital status** (married = 1, not married = 0)	1.3	0.2	2.0	0.0	1.0	1.7
_cons	0.1	0.0	−13.5	0.0	0.1	0.2
Log pseudo likelihood = −14,632,786						
Number of obs = 15,349						
Wald chi2(15) = 135.0						
Prob > chi2 = 0.000						
Pseudo R2 = 0.013						

R = Reference category

Source: Authors’ computation based on the UNHS 2016/17 data

### Impoverishment

The results in Tables 5 and 6 show the impoverishing effects of OOP payments in Uganda using the international poverty line (US$1.91 per day) and the Uganda national poverty line respectively. The results show that OOP increase the incidence and depth/intensity of poverty among the poor. The pattern is similar for all the poverty lines considered. A decrease in the impoverishment headcount was observed from 2005/06 through to 2016/17, although the decline in the impoverishment headcount between 2012/13 and 2016/17 is minimal.

**Table 5:** Impoverishment indicators using the international poverty line

	Pre-payment poverty (%) (A)	Post-payment poverty (%) (B)	Absolute difference (%) (B-A)
**2005/06 (PPP = 513.9492)**			
Poverty headcount	51.8	57.0	5.2
Normalised mean positive poverty gap	35.2	37.0	
**2009/10 (PPP = 741.3262)**			
Poverty headcount	46.3	50.8	4.5
Normalised mean positive poverty gap	33.4	34.9	
**2012/13 (PPP = 1043.083)**			
Poverty headcount	64.0	67.2	3.2
Normalised mean positive poverty gap	39.4	40.2	
**2016/17 (PPP = 1161.989)**	51.8	57.0	5.2
Poverty headcount	35.2	37.0	
Normalised mean positive poverty gap			

Source: Authors’ computation based on the UNHS 2005/06-2016/17 data

**Table 6:** Impoverishment indicators using Uganda’s national poverty line

	Pre-payment poverty (%) (A)	Post-payment poverty (%) (B)	Absolute difference (%) (B-A)
**2005/06**			
Poverty headcount	31.1	35.6	4.6
Normalised mean positive poverty gap	35.2	37.0	
**2009/10**			
Poverty headcount	23.2	27.2	4.0
Normalised mean positive poverty gap	27.6	28.3	
**2012/13**			
Poverty headcount	19.7	21.7	2.0
Normalised mean positive poverty gap	26.4	26.7	
**2016/17**			
Poverty headcount	21.5	24.1	2.7
Normalised mean positive poverty gap	5.3	6.0	

Source: Authors’ computation based on the UNHS 2005/06-2016/17 data

Table 7 shows the disaggregation of impoverishment effect by socio-economic status, residence and region. The results show that the impoverishment effect is mainly concentrated in the middle and second richest quintiles of socio-economic status. It is also largely concentrated in the Central and Western regions. The distribution of impoverishment by residence is less clear-cut, showing a mixed pattern over the different years considered.

**Table 7:** Disaggregation of impoverishment headcount

Disaggregation variable	2005/06	2009/10	2012/13	2016/17
**Total**	5.2	4.5	3.2	2.7
**Socio-economic status quintiles**				
Poorest	0.0	0.0	0.0	0.0
Second poorest	0.0	0.0	0.0	0.0
Middle	16.7	18.6	0.0	11.0
Second richest	8.3	2.5	14.9	2.5
Richest	1.0	1.5	1.2	0.0
**Residence**				
Rural	5.6	4.9	2.9	3.1
Urban	2.9	2.2	4.1	1.6
**Region**				
Central	5.4	3.4	4.8	2.5
Eastern	5.2	5.3	2.0	2.5
Northern	2.7	4.4	2.7	3.0
Western	6.9	4.9	3.4	3.0

Source: Authors’ computation based on the UNHS 2005/06-2016/17 data

## Discussion

This study set out to assess the trends in the status of financial protection in Uganda with a view of informing strategies for strengthening financial risk protection. The findings show that Ugandans still lack financial risk protection, and there has been a reversal of the trend in catastrophic expenditure and impoverishment rates. This pattern threatens Uganda’s ability to attain UHC. This pattern is not surprising especially in the context of the country’s dependence on OOP payments, which requires urgent attention. The main strength of this study is that unlike all the previous studies which showed a snapshot analysis of the situation of financial risk protection at a point in time, this study was able to show a trend over time. This paper also shows equity aspects by disaggregating the financial risk protection indicators using various equity dimensions. It also shows factors that are associated with households facing financial risk due to direct OOP payments.

The results from this study are consistent with previous assessments of financial risk protection in Uganda and other low-income countries that depend heavily on direct OOP payments [15, 16, 22]. However, when we compare the results of Uganda to similar studies in Kenya [23, 24], Rwanda, Zambia, South Africa, Tanzania and Ghana [25–27], Uganda’s estimated levels of catastrophic payments and impoverishment are higher in magnitude than all these countries. However, the results are consistent with literature as countries, where the contribution of OOP payments in total health expenditure is higher, are more likely to have higher levels of financial catastrophe or impoverishment from OOP spending. The main difference between Uganda and the other countries is that it has a much higher level of OOP payments (at 40%). In addition, these countries have significant prepayment from prepayment schemes that compliments the general tax contributions to health paid through government budgets.

The results of this study provide important implications for policymakers in Uganda. The fact that there could be a reversal in the gains observed in the reduction of financial risk highlights the importance of continuous monitoring. It also implies that there is a need to move away from the business as usual approach in Uganda. Although Uganda established free care policy for primary health care services by abolishing user fees, the allocation of resources to the health sector from the national budget has not matched the need. Establishing a well-intentioned policy mandate without adequately funding it may produce reverse results as is being experienced in Uganda where OOP payments have continued to increase even in the context of no user fees [7]. To highlight the extent of underfunding, whereas, consumer price index published by the UBOS shows that the price of consumables/utilities has increased by over 20% in the previous decade; the allocation to health facilities purchase of these has been stagnant (reduced in real terms when adjusted for inflation and exchange rate depreciation) [28]. This results in the lack of critical inputs required to provide quality health care in the public sector, leading to the private sector providing the majority of services [29]. This has led to inequitable access to services, as only those who can pay access services [30]. However, even for the non-poor who can pay for services within the private sector, this approach is not sustainable in the long-term as they may be impoverished.

Increasing public financing would enable reduced exposure to financial risk, especially among the poor who pay OOP because of the limited availability of services in the public sector. Furthermore, one of the ways Uganda can reduce reliance on OOP payments is through moving towards mandatory health insurance. Uganda should fast track its plans for establishing a single pool mandatory national health insurance scheme so as to enable strategic purchasing of health care services and reduce direct OOP health payment at the point of care. However, this should be done concurrently with quality improvement interventions. It has also been shown in other countries that well-designed supply interventions aimed at improving quality of care are protective against OOP payments and have operational simplicity and greater provider accountability [31]. Moreover, the design of the insurance scheme also matters. It is recommended that that in a context such as Uganda, the a national health insurance scheme should mainly be funded through general taxes combined with adequately regulated insurance premiums as opposed to employment based contributions (i.e. labour taxes) [32].

This study is not without any limitations. The main limitation is the absence of information on access/utilization of services across the population for how financial risk was measured. The goal of UHC is to enable access to all who need care while minimizing the extent to financial risk. By not being able to show the extent to access, this paper did not show whether the lack of financial risk protection was influenced by the level of (or the lack of) access. Some additional limitations arise from the data used. Although the UNHS has critical data useful for this analysis, it has some major gaps that if addressed, would enable the survey to provide more useful information for decision-makers. For instance, one dimension that could be useful is to identify the type of service (i.e. inpatient or outpatient) used and rate of utilization to identify the drivers of OOP payments for policy targeting.

## Conclusion

In this study, we present empirical evidence on the extent of financial risk protection in health in Uganda. The financial burden due to OOP payments remains high and there is a risk of a reversal of previous gains in reducing this burden. We show that some households are more vulnerable to incurring the burden of OOP health payments. The study shows that Uganda needs to reconsider its strategies to decrease the burden of OOP payments. In particular, there is a need to fast-track the design and implementation of the mechanisms for protecting the population from financial catastrophe and impoverishment, especially the planned mandatory health insurance scheme. This should be done together with interventions aimed at improving effective coverage of quality health care in the public sector facilities. Lastly, monitoring financial risk protection should be institutionalised as part of monitoring the implementation of health financing reforms in Uganda.

## Data Availability

The datasets analyzed in the current study are available from the Uganda Bureau of Statistics. UNHS data is available on the UBOS website (http://www.ubos.org/unda/index.php/catalog/51). Data analysis files and other materials can be obtained from the corresponding author.

## Abbreviations

DFID: Department for International Development of United Kingdom
OOP: Out of Pocket Payments
PPP: Purchasing Power Parity
SDGs: Sustainable Development Goals
UBOS: Uganda Bureau of Statistics
UHC: Universal Health Coverage
UNHS: Uganda National Household Survey
WB: World Bank
WHO: World Health Organisation

## Footnotes

1 The PPP conversion rates for the different years surveys are; 2005/06 (PPP = 513.9492), 2009/10 (PPP = 741.3262), 2012/13 (PPP = 1043.083) and 2016/17 (PPP = 1161.989)
