# Correction to: What has been the progress in addressing financial risk in Uganda? Analysis of catastrophe and impoverishment due to health payments

**DOI:** 10.1186/s12913-020-05707-3

**Published:** 2020-09-08

**Authors:** Brendan Kwesiga, Tom Aliti, Pamela Nabukhonzo, Susan Najuko, Peter Byawaka, Justine Hsu, John E. Ataguba, Grace Kabaniha

**Affiliations:** 1 World Health Organization, Health Systems Cluster, Nairobi, Kenya; 2 Ministry of Health, Planning Department, Kampala, Uganda; 3 Uganda Bureau of Statistics, Kampala, Uganda; 4 World Health Organization, Economic Analysis Cluster, Geneva, Switzerland; 5 Health Economics Unit, School of Public Health and Family Medicine, University of Cape Town, Cape Town, South Africa; 6 World Health Organization, Health Systems Cluster India Country Office, New Delhi, India

**Correction to: BMC Health Serv Res 20, 741 (2020)**

**https://doi.org/10.1186/s12913-020-05500-2**

Following publication of the original article [1], the authors identified some errors in Table 5 and Table 6. The correct tables are given below.

**Table 5:** Impoverishment indicators at $1.91 per day poverty line –PPP

	Pre-payment poverty (%) (A)	Post-payment poverty (%) (B)	Absolute difference (%) (B-A)
**2005/06 (PPP = 513.9492)**			
Poverty headcount	51.8	57.02	5.2
Normalized mean positive poverty gap	35.2	37.0	
**2009/10 (PPP = 741.3262)**			
Poverty headcount	46.3	50.8	4.5
Normalized mean positive poverty gap	33.39	34.9	
**2012/13 (PPP = 1043.083)**			
Poverty headcount	64.0	67.2	3.2
Normalized mean positive poverty gap	39.4	40.2	
**2016/17 (PPP = 1161.989)**			
**Poverty headcount**	**49.0**	**51.7**	**2.7**
**Normalized mean positive poverty gap**	**16.7**	**18.1**	

Source: Authors’ computation based on the UNHS 2005–2017 data

**Table 6:** Impoverishment indicators (Uganda’s national poverty line)

	Pre-payment poverty (%) (A)	Post-payment poverty (%) (B)	Absolute difference (%) (B-A)
**2005/06**			
Poverty headcount	31.1	35.6	4.6
Normalized mean positive poverty gap	35.2	37.0	
**2009/10**			
Poverty headcount	23.2	27.2	4.0
Normalized mean positive poverty gap	27.6	28.3	
**2012/13**			
Poverty headcount	19.7	21.7	2.0
Normalized mean positive poverty gap	26.4	26.7	
**2016/17**			
Poverty headcount	21.5	24.1	**2.5**
Normalized mean positive poverty gap	5.3	6.0	

Source: Authors’ computation based on the UNHS 2005–2017 data

## References

[1] Kwesiga (2020). BMC Health Serv Res.

